# Nomograms for predicting the overall and cancer-specific survival of patients with classical Hodgkin lymphoma: a SEER-based study

**DOI:** 10.18632/oncotarget.21722

**Published:** 2017-10-09

**Authors:** Yue Zhang, Juan Zhang, Hui Zeng, Xiao-Huan Zhou, He-Bing Zhou

**Affiliations:** ^1^ Department of Hematology, Beijing Luhe Hospital, Capital Medical University, Tongzhou, Beijing 101149, People's Republic of China

**Keywords:** classical Hodgkin lymphoma, nomogram, overall survival, cancer-specific survival

## Abstract

The aim of this study was to establish nomograms, based on significant clinicopathologic parameters, for predicting the overall survival (OS) and the cancer-specific survival (CSS) of patients with classical Hodgkin lymphoma (CHL). The data of 43,330 CHL patients, diagnosed between 1983 and 2014, were obtainedfrom the database of the Surveillance, Epidemiology, and End Results (SEER) program. These patients were randomly divided into training (n = 30,339) and validation (n = 12,991) cohorts. The Kaplan-Meier method and Cox proportional hazards regression model were used to evaluate the prognostic effects of multiple clinicopathologic parameters on survival. Significant prognostic factors were combined to build nomograms. The predictive performance of nomograms was evaluated using the index of concordance (C-index) and calibration curves. In the training cohort, on univariate and multivariate analyses, age at diagnosis, gender, race, Ann Arbor stage, and histological type significantly correlated with the survival outcomes. These characteristics were used to establish nomograms. The nomograms showed good accuracy in predicting 1-, 5-, and 10-year OS and CSS, with a C-index of 0.794 (95% confidence interval [CI], 0.789-0.799) for OS and 0.760 (95% CI, 0.753-0.767) for CSS. In the validation cohort, the C-index for nomogram-based predictions was 0.787 (95% CI, 0.779-0.795) for OS and 0.769 (95% CI, 0.758-0.780) for CSS. All calibration curves revealed excellent consistency between predicted and actual survival. In summary, novel nomograms were established and validated to predict OS and CSS for patients with CHL. These new prognostic models could aid in improved prediction of survival outcomes leading to reasonable treatment recommendations.

## INTRODUCTION

At present, Hodgkin lymphoma (HL) is a treatable malignancy for most patients. In economically developed countries, HL presents a bimodal incidence curve with peaks at both 15-30 years of age and over 55 years of age [1]. A 2017 report indicates that in the United States, approximately 8,260 people were diagnosed with HL, with an estimated 1,070 reported deaths from the disease [2]. The 2008 WHO classification criteria recognize two histologic types of HL: “nodular lymphocyte predominant”, which accounts for 5% of cases, and “classic” HL (CHL), which accounts for about 95% of cases. The latter type consists of four subtypes: “lymphocyte-rich”, “mixed cellularity”, “lymphocyte depletion”, and “nodular sclerosis” [3].

In recent years, significant progress has been made in the treatment of patients with HL, and more than 80% of patients have been cured by front-line therapy [4–6]. However, along with the significant increases in the cure rate of HL, there have also been increases in treatment-related long-term toxicities, especially for patients diagnosed with early and medium-stage disease. Conversely, patients with refractory or relapsed disease still have a poor prognosis [7]. Therefore, an accurate prognostic model for predicting survival is needed to reduce over-treatment in low-risk patients, and to guide treatment selection for high-risk patients.

To date, studies have reported that many clinical, histopathological, and laboratory parameters are prognostic factors in this disease [8–10]. HL is commonly staged using the Ann Arbor system. The staging system depends on the region of the malignant tissue and the systemic symptoms of the lymphoma, and it has approximately the same clinical role as the TNM staging of solid tumors [11, 12]. Based on the presence of adverse disease factors, HL patients can be divided into three groups: favorable early stage (stage I-II with no adverse factors), unfavorable early stage (stage I-II with any of the adverse factors), and advanced stage (stage III-IV). Adverse factors include B symptoms, enlarged mediastinal lymph nodes, extranodal involvement, and higher erythrocyte sedimentation rates [13–15]. The most widely used clinical indicator of risk in HL is the International Prognostic Score (IPS), which is based on the number of unfavorable prognostic factors that are present, such as age ≥ 45 years, male gender, Ann Arbor stage IV, serum albumin < 4 g/dL, hemoglobin < 10.5 g/dL, white blood cell count ≥ 15,000/mm^3^, and lymphocyte count < 600/mm^3^ [16]. However, the IPS is only beneficial for providing guidance about the clinical strategies and prognosis of stage III-IV patients. In addition, these prognostic systems do not take into consideration the histopathological parameters, which could affect survival rates. Neglecting histopathological parameters or other prognostically significant features may reduce the accuracy of survival predictions. Therefore, an improved prognostic evaluation system that includes histopathology and host status is needed in clinical practice.

A nomogram is a graphical representation of a mathematical model, in which information on several characteristics is combined to predict a specific endpoint. A convenient graphical representation in the form of a nomogram allows predictions to be obtained easily and quickly in practice [17]. By integrating various important factors, a nomogram can provide individualized estimates of the probability of an event, such as a patient's individual probability of disease recurrence or death. Therefore, the nomogram has become a reliable tool for predicting the clinical outcomes of many types of cancer [18–21].

However, the published literature does not include any nomogram that uses available prognostic factors to predict survival outcomes in patients with CHL. In this study, patient records from the Surveillance, Epidemiology, and End Results (SEER) cancer registries were used to identify risk factors associated with the overall survival (OS) and cancer-specific survival (CSS) of CHL patients. The SEER data were used to establish nomograms that allow graphic-based predictions of the prognosis of these patients.

## RESULTS

### Clinicopathologic characteristics of the patients

A total of 46,602 CHL patients were identified in the SEER database for this study. Of these, 3272 patients were excluded because of missing data, leaving 43,330 patients for inclusion in our analyses. Patients were randomly divided into a training cohort (n = 30,339) and a validation cohort (n = 12,991). The clinicopathologic characteristics of patients available from the SEER database in the training and validation cohorts are summarized in Table 1. There were no substantive differences between the two cohorts.

**Table 1:** Characteristics of the training and validation cohorts

Characteristic	Training cohort (n = 30339)		Validation cohort (n = 12991)	
	No.	%	No.	%
Age at diagnosis, years				
Median ± SD	36 ± 20.0		36 ± 20.1	
Range	2 - 99		1 - 98	
Gender				
Male	16478	54.3	7044	54.2
Female	13861	45.7	5947	45.8
Race				
White	25518	84.1	10951	84.3
Black	3205	10.6	1382	10.6
Others^a^	1616	5.3	658	5.1
Ann Arbor stage				
Stage I/II	18054	59.5	7734	59.5
Stage III/IV	12285	40.5	5257	40.5
Histological type				
LR	1048	3.5	443	3.4
MC	4536	15.0	1924	14.8
LD	471	1.5	209	1.6
NS	18565	61.2	7924	61.0
NOS	5719	18.8	2491	19.2

^a^Others includes American Indian/Alaskan Native and Asian/Pacific Islander.

Abbreviations: SD, standard deviation; LR, lymphocyte-rich; MC, mixed cellularity; LD, lymphocyte-depleted; NS, nodular sclerosis; NOS, not otherwise specified.

### OS and CSS in the training cohort

The median OS was 83 months (range, 1 to 383 months), and the 1-, 5-, and 10-year OS rates were 89.7%, 79.8%, and 72.3%, respectively. The median CSS was 80 months (range, 1 to 383 months), and the 1-, 5-, and 10-year CSS rates were 93.5%, 86.6%, and 83.3%, respectively.

### Independent prognostic factors in the training cohort

**Figure 1:**
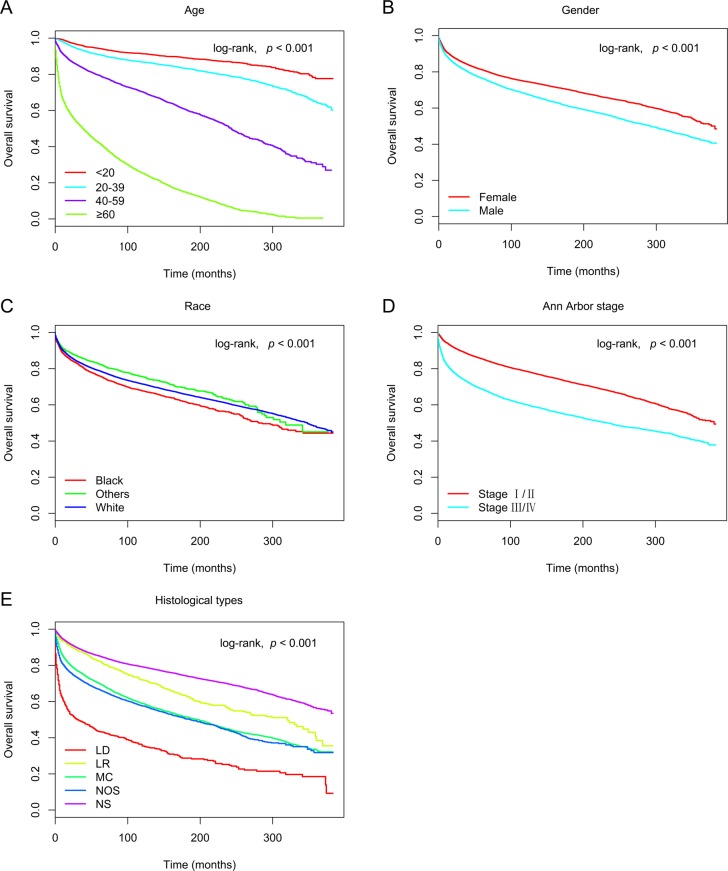
Kaplan-Meier survival curves for overall survival in the training cohort, as stratified by **(A)** age (log-rank, χ^2^ = 1.14E+04, *p* < 0.001), **(B)** gender (log-rank, χ^2^ = 2.08E+02, *p* < 0.001), **(C)** race (log-rank, χ^2^ = 3.24E+01, *p* < 0.001), **(D)** Ann Arbor stage (log-rank, χ^2^ = 1.37E+03, *p* < 0.001), and **(E)** histological type (log-rank, χ^2^ = 2.05E+03, *p* < 0.001). Abbreviations: LD, lymphocyte-depleted; LR, lymphocyte-rich; MC, mixed cellularity; NOS, not otherwise specified; NS, nodular sclerosis.

**Figure 2:**
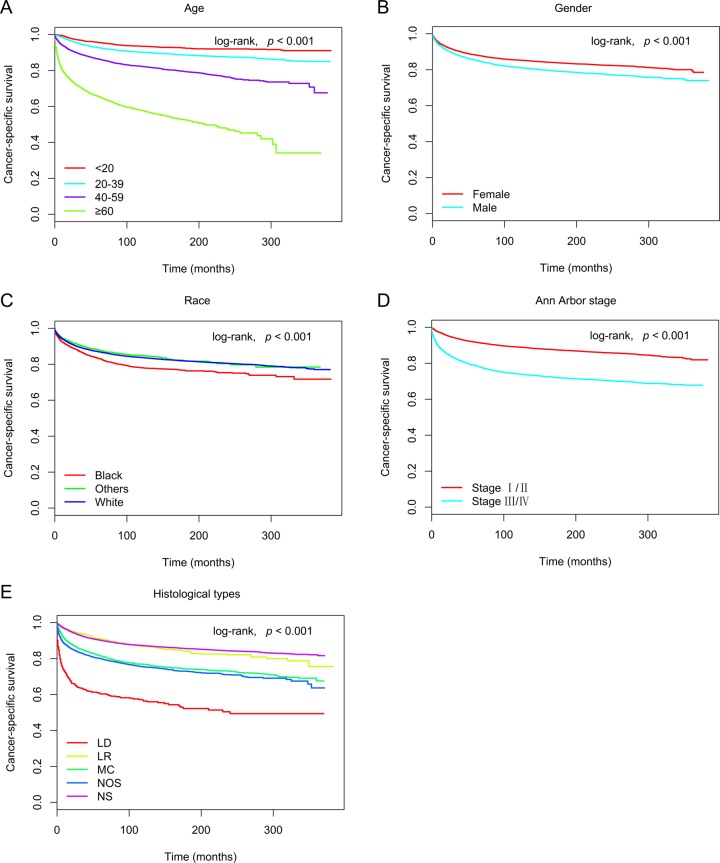
Kaplan-Meier survival curves for cancer-specific survival in the training cohort, as stratified by **(A)** age (log-rank, χ^2^ = 3.36E+03, *p* < 0.001), **(B)** gender (log-rank, χ^2^ = 7.98E+01, *p* < 0.001), **(C)** race (log-rank, χ^2^ = 5.24E+01, *p* < 0.001), **(D)** Ann Arbor stage (log-rank, χ^2^ = 1.00E+03, *p* < 0.001), and **(E)** histological type (log-rank, χ^2^ = 9.50E+02, *p* < 0.001). Abbreviations: LD, lymphocyte-depleted; LR, lymphocyte-rich; MC, mixed cellularity; NOS, not otherwise specified; NS, nodular sclerosis.

In the training cohort, 30,339 patients were included in univariate and multivariate analyses to determine predictors of OS and CSS. As shown in Figure 1 and Figure 2, age at diagnosis, gender, race, Ann Arbor stage, and histological type remarkably correlated with OS and CSS in univariate survival analyses using the Kaplan-Meier method, and were further compared using the log-rank test (*p* < 0.05). Cox proportional hazards regression modeling was used to further explore the influences of all variables. The multivariate analyses of OS and CSS showed elevated hazard ratios (HRs) for the following characteristics: older age, male gender, black race, stage III/IV, and the lymphocyte-depleted histological type (*p* < 0.05) (Table 2).

**Table 2:** Multivariate analysis of overall survival and cancer-specific survival in the training cohort

Variable	OS			CSS		
	HR	95% CI	*P*	HR	95% CI	*P*
Age at diagnosis, years						
< 20	ref			ref		
20-39	1.603	1.443 to 1.781	< 0.001	1.521	1.334 to 1.735	< 0.001
40-59	3.885	3.498 to 4.316	< 0.001	2.802	2.455 to 3.199	< 0.001
≥ 60	13.845	12.493 to 15.343	< 0.001	7.473	6.565 to 8.507	< 0.001
Gender						
Male	1.259	1.205 to 1.316	< 0.001	1.191	1.121 to 1.264	< 0.001
Female	ref			ref		
Race						
White	ref			ref		
Black	1.358	1.269 to 1.454	< 0.001	1.481	1.357 to 1.616	< 0.001
Others^a^	0.926	0.830 to 1.033	0.170	1.056	0.919 to 1.215	0.441
Ann Arbor stage						
Stage I/II	ref			ref		
Stage III/IV	1.705	1.632 to 1.781	< 0.001	2.058	1.937 to 2.187	< 0.001
Histological type						
LR	ref			ref		
MC	1.401	1.237 to 1.587	< 0.001	1.654	1.367 to 2.001	< 0.001
LD	2.305	1.959 to 2.713	< 0.001	3.146	2.493 to 3.972	< 0.001
NS	1.109	0.982 to 1.251	0.096	1.338	1.112 to 1.610	0.002
NOS	1.535	1.355 to 1.739	< 0.001	1.814	1.501 to 2.192	< 0.001

^a^Others includes American Indian/Alaskan Native and Asian/Pacific Islander.

Abbreviations: OS, overall survival; CSS, cancer-specific survival; HR, hazard ratio; CI, confidence interval; LR, lymphocyte-rich; MC, mixed cellularity; LD, lymphocyte-depleted; NS, nodular sclerosis; NOS, not otherwise specified; ref, reference.

### Prognostic nomograms for OS and CSS

**Figure 3:**
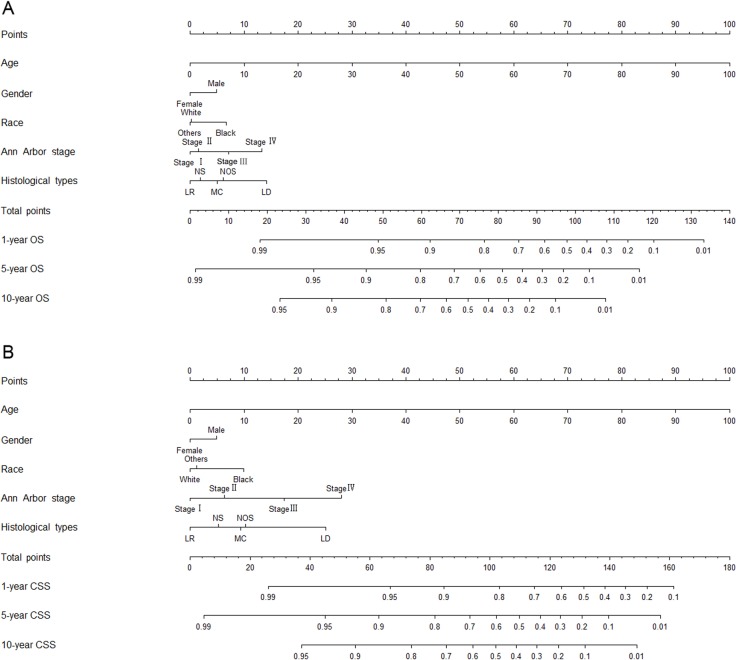
Nomograms for predicting the 1-, 5-, and 10-year **(A)** overall survival and **(B)** cancer-specific survival of classical Hodgkin lymphoma patients. Abbreviations: OS, overall survival; CSS, cancer-specific survival; LD, lymphocyte-depleted; LR, lymphocyte-rich; MC, mixed cellularity; NOS, not otherwise specified; NS, nodular sclerosis.

The prognostic nomograms included all the significant independent factors in Cox proportional hazards regression in the training cohort. The prognostic nomogram for 1-, 5-, and 10-year OS is shown in Figure 3A. The prognostic nomogram for 1-, 5-, and 10-year CSS is shown in Figure 3B. By adding up the scores associated with each variable, and projecting total scores to the bottom scale, probabilities can be estimated for 1-, 5-, and 10-year OS and CSS.

In general, both the OS and CSS rates were better for women, relatively younger patients, patients with Ann Arbor stage I/II disease, patients of non-black race, and patients with the lymphocyte-rich histological type of CHL. With the aid of nomograms, it was possible to effectively predict prognosis according to individual patient characteristics.

### Validation of the nomograms

**Figure 4:**
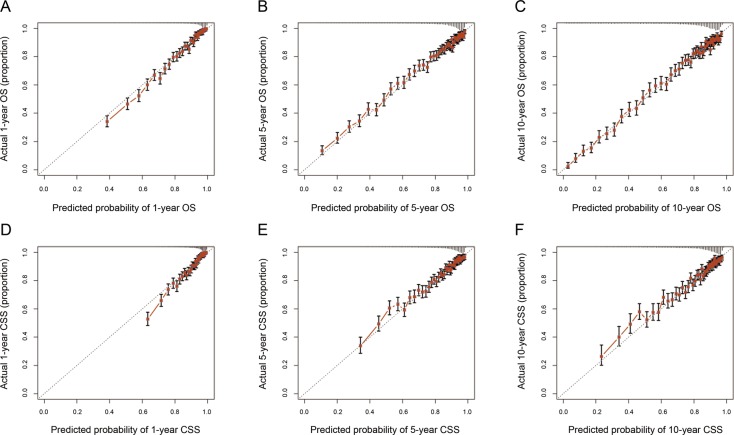
The calibration curves for predictions of overall survival **(A-C)** and cancer-specific survival **(D-F)** in the training cohort at 1, 5, and 10 years after diagnosis. The dashed line represents perfect agreement between the nomogram-predicted probability (x-axis) and the actual probability calculated from a Kaplan-Meier analysis (y-axis). A perfectly accurate nomogram prediction model would result in a plot where the actual and predicted probabilities, for the given groups, fall along the 45° line. The distance between the pairs and the 45° line is a measure of the absolute error of the nomogram's prediction. Abbreviations: OS, overall survival; CSS, cancer-specific survival.

**Figure 5:**
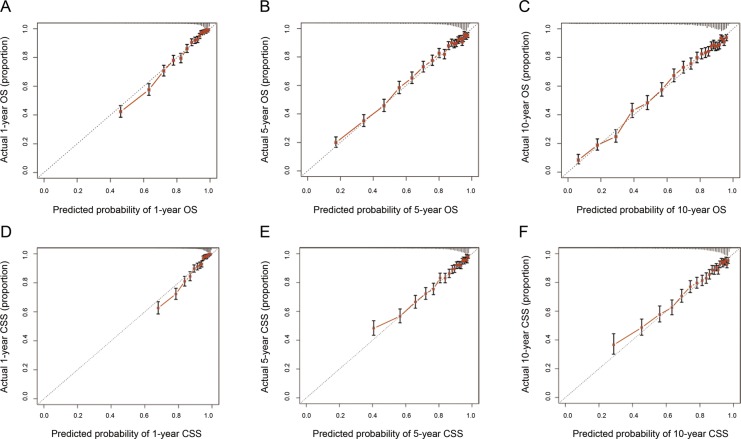
The calibration curves for predictions of overall survival **(A-C)** and cancer-specific survival **(D-F)** in the validation cohort at 1, 5, and 10 years after diagnosis. The dashed line represents perfect correspondence between the nomogram-predicted probability (x-axis) and the actual probability calculated from a Kaplan-Meier analysis (y-axis). A perfectly accurate nomogram prediction model would result in a plot where the actual and predicted probabilities, for the given groups, fall along the 45° line. The distance between the pairs and the 45° line is a measure of the absolute error of the nomogram's prediction. Abbreviations: OS, overall survival; CSS, cancer-specific survival.

Validation of nomograms was performed using bootstrap analyses with 1000 resamples, processed both internally and externally. Analysis of the internal validation cohort (training cohort) showed C-index values of 0.794 (95% CI, 0.789-0.799) for nomogram predictions of OS and 0.760 (95% CI, 0.753-0.767) for nomogram predictions of CSS. Similarly, in the external validation cohort, the C-index values for predicting OS and CSS were 0.787 (95% CI, 0.779-0.795) and 0.769 (95% CI, 0.758-0.780), respectively (Table 3). These findings indicate that the nomogram models were reasonably accurate. The internal and external calibration curves demonstrated excellent agreement between predicted and observed values of 1-, 5-, and 10-year OS and CSS, in both the training and validation cohorts (Figure 4 and Figure 5).

**Table 3:** C-indexes for the nomogram to predict overall survival and cancer-specific survival

Group	OS		CSS	
	C-index	95% CI	C-index	95% CI
Training cohort	0.794	0.789 to 0.799	0.760	0.753 to 0.767
Validation cohort	0.787	0.779 to 0.795	0.769	0.758 to 0.780

Abbreviations: OS, overall survival; CSS, cancer-specific survival; C-index, index of concordance; CI, confidence interval.

## DISCUSSION

The nomogram is a graphic representation of a mathematical model that combines biological and clinical variables to determine the probabilities of clinical events. Nomograms are widely used in medicine. Some researchers have analyzed the survival outcomes of different tumors, using the SEER database. Compared to the current tumor staging system, the nomogram showed better prediction accuracy and prognostic value [22–25].

As the SEER program consists of 18 cancer registries, covering almost thousands of hospitals and nearly 30% of the country's total population, the wide range of data included in our nomograms should allow the nomograms to be used widely for decision making in clinical practice.

Considering the relatively long-term survival potential of HL, deaths in patients with HL often occur because of non-HL causes. Thus, OS may not provide an accurate reflection of the long-term survival implications of HL. Therefore, taking other causes of death into consideration is necessary when estimating the CSS of HL.

In the present study, nomograms were developed based on 30,339 cases from the SEER database. The nomograms were used to predict the 1-, 5-, and 10-year OS and CSS rates of patients with CHL, based on five significant factors: age at diagnosis, gender, race, Ann Arbor stage, and histological type. The objective was to effectively and visually predict prognosis from specific patient characteristics. The discriminative performance of the nomograms was evaluated using an internal bootstrap resampling method. The C-index demonstrated the capacity of nomograms to predict the 1-, 5-, and 10-year OS and CSS rates of CHL patients.

As shown in the nomograms that we have presented, age at diagnosis had a strong prognostic association with OS and CSS. On an average, a 60-year old patient had 5-year OS and CSS rates reduced by 50% and 41%, as compared to a 30-year old patient who had the same exposure to other risk factors. Age has been demonstrated to be a significant predictive and prognostic factor in previous studies too [8, 26].

The different histological subtypes of HL are associated with significant biological and prognostic differences [27–30]. Therefore, in the present study, histological type was added to the prognostic factors that were included in nomograms. In the SEER data, nearly one in five patients had a histological type of “not otherwise specified” (NOS). Sally et al. suggested that an increase in the proportion of NOS in recent years is related to the prevalence of non-excisional biopsies, with an insufficiency of biopsy specimens for histologic diagnosis. Although NOS lacks a biological definition, it should be included as a subtype in HL research, since it is the second most common CHL category [31]. Therefore, our nomogram prognostic models included the NOS histological type.

As shown in the nomograms, histological type, which is not included in current staging systems, was an important predictive factor of OS and CSS. Therefore, we suggest the inclusion of histological type in future CHL prognostic evaluation systems.

This study has several limitations. First, as a retrospective study, it is subject to inherent, unavoidable biases. Thus, to confirm the results, large randomized controlled studies may be required. Second, data on important therapies, such as chemotherapy and radiation therapy, were not accessible in the SEER database. Finally, there are many other factors that may influence prognosis, such as B symptoms, extranodal involvement, and some molecular markers. The SEER cancer registry did not provide information about these factors, and these potential prognostic factors were therefore not included in the nomogram. Despite these limitations, the present study is the first to apply nomogram model to predict the survival of CHL patients.

The present study showed that age, gender, race, Ann Arbor stage, and histological types were independent risk factors for survival in patients with CHL. Nomograms were developed to accurately predict the 1-, 5-, and 10-year OS and CSS rates of these patients, based on patient-specific characteristics. These predictive tools could help clinicians identify high-risk patients and obtain more precise evaluations of patient survival.

## MATERIALS AND METHODS

### Data source

Data used in this study were retrieved from the SEER registry database of the National Cancer Institute. The SEER database is a collection of information about cancer incidence, prevalence, mortality, population-based variables, primary tumor characteristics, and treatment in 18 registries within the United States (http://seer.cancer.gov/).

### Study population

Data for patients diagnosed between 1983 and 2014 were examined using SEER^*^Stat software (Version 8.3.2). Patients with CHL were included in this analysis. The following information was obtained for each patient: year of diagnosis, age at diagnosis, gender, race, Ann Arbor stage, histological type, survival information, and cause of death. Patients with missing data on any of these characteristics were excluded. A total of 43,330 CHL patients were randomized to two groups (training cohort, n = 30,339 and validation cohort, n = 12,991). Patients whose race was recorded as American Indian/Alaskan Native or Asian/Pacific Islander in SEER were assigned to an “others” race category for analysis.

### Construction of the nomograms

A training cohort was used to establish nomograms. One of our primary endpoints of interest was OS, which was defined as the time from diagnosis to death from any cause. In the analysis of OS, patients who were alive at the last follow-up were counted as censored observations. The other primary endpoint of interest was CSS, which was defined as the time from diagnosis to death attributed to CHL. In the analysis of CSS, patients who died of other causes or were alive at the last follow-up were counted as censored observations.

The Kaplan-Meier method and Cox proportional hazards regression model were used to determine survival-related factors. The factors that were observed to have significant associations with survival in univariate or multivariate analyses (*P* < 0.05) were used to build the nomograms for OS and CSS.

### Validation of the nomograms

Nomograms were subjected to 1000 bootstrap resamples for internal validation in the training cohort and external validation in the validation cohort, respectively. Marginal estimates and model-average prediction probabilities were used to create calibration curves. In a perfectly calibrated model, the predictions should fall on the diagonal 45° line of the calibration plot. Predictive performance was assessed using the C-index, which resembles the area under the curve (AUC), but appears to be better-suited for censored data [32]. Unlike the maximum value of the C-index of 0.5, which indicates a random chance to correctly discriminate outcome with the model, the maximum value of the C-index obtained was 1.0, indicating a perfect discrimination. A larger C-index indicates more accurate prognostic predictions [33].

### Statistical analysis

The statistical analysis was performed using IBM SPSS statistics 22 software (SPSS Inc, Chicago, IL, USA) and R version 3.2.2 software (Institute for Statistics and Mathematics, Vienna, Austria; http://www.r-project.org/). The “rms” R library (cran.r-project.org/web/packages/rms) was used to construct survival models [34]. *P* values were two-sided and *P* < 0.05 was regarded as indicating statistical significance.

### Ethical statement

According to the guidelines of the government of the United States, data released through the SEER database does not require informed patient consent.

## Abbreviations

CHL: classical Hodgkin lymphoma
CI: confidence interval
CSS: cancer-specific survival
HL: Hodgkin lymphoma
HR: hazard ratio
IPS: International Prognostic Score
LD: lymphocyte-depleted
LR: lymphocyte-rich
MC: mixed cellularity
NOS: not otherwise specified
NS: nodular sclerosis
OS: overall survival
SEER: Surveillance
